# Liuzijue training improves hypertension and modulates gut microbiota profile

**DOI:** 10.3389/fcvm.2023.1075084

**Published:** 2023-01-25

**Authors:** Sha Wu, Caiping Zheng, Nannan Liu, Tingting Deng, Jie Wang, Luming Qi, Lina Xia

**Affiliations:** ^1^State Administration of Traditional Chinese Medicine, Key Laboratory of Traditional Chinese Medicine Regimen and Health, Chengdu University of Traditional Chinese Medicine, Chengdu, Sichuan, China; ^2^School of Health Preservation and Rehabilitation, Chengdu University of Traditional Chinese Medicine, Chengdu, Sichuan, China; ^3^Key Laboratory of Traditional Chinese Medicine Regimen and Health of Sichuan Province, Chengdu, Sichuan, China; ^4^Jincheng People's Hospital, Jincheng, Shanxi, China; ^5^Nursing College of Chengdu University of Traditional Chinese Medicine, Chengdu, Sichuan, China

**Keywords:** Liuzijue exercise, blood pressure, hypertension, immune homeostasis, gut microbiota

## Abstract

**Background:**

Liuzijue training (LZJ) is a traditional exercise integrating breathing meditation and physical exercise, which could prevent and improve hypertension symptoms.

**Purpose:**

We aimed to evaluate the therapeutic effect of LZJ on hypertensive patients from the perspectives of blood pressure (BP), vascular endothelial function, immune homeostasis, and gut microbiota.

**Methods:**

We conducted a randomized, controlled, single-blind experiment to assess the effect of 12 weeks LZJ in hypertensive patients. We measured the blood pressure level, vascular endothelial function, serum inflammatory factor concentration, and fecal microbial composition of hypertension patients.

**Results:**

Compared with aerobic training, LZJ has a more significant effect on serum inflammatory factors (IL-6 and IL-10) and gut microbiota. PCoA analysis showed that LZJ tended to transform the gut microbiota structure of hypertensive subjects into that of healthy people. This process involves significant changes in Bacteroides, Clostridium_sensu_stricto_1, Escherichia-Shigella, Haemophilus, Megamonas, and Parabacteroides. In particular, Bacteroides and Escherichia-Shigella, these bacteria were closely related to the improvement of BP in hypertensive patients.

**Conclusion:**

In conclusion, our results confirm that LZJ could be used as an adjuvant treatment for hypertensive patients, which could effectively reduce BP, improve the immune homeostasis and gut microbiota structure in patients, and provide a theoretical reference for the use of LZJ in the clinic.

**Clinical trial registration:**

http://www.chictr.org.cn/listbycreater.aspx, identifier: ChiCTR2200066269.

## 1. Introduction

Hypertension is a common chronic disease caused by the interaction of genetic and environmental factors (1, 2). It is always accompanied by the damage of vascular endothelium that will cause severe target organ damage in the later stage, with high morbidity and mortality rates (3, 4). At present, oral antihypertensive drugs are the main strategy to treat hypertension in the clinical. These drugs have the advantage of being powerful and effective in lowering blood pressure (BP) (5, 6). However, they can not prevent the development of hypertensive comorbidities. Previous studies have shown that many patients stop taking medicines due to their adverse effects (7). Searching for more comprehensive and effective treatments is one of the world's major public health problems to treat this disease.

Numerous studies have shown that regular exercise could reduce BP levels and improve its complications (8). The guidelines for the prevention and treatment of hypertension advocate that aerobic training (AT) should be used as adjuvant therapy for lowering BP (9). Traditional Chinese health exercise is a feature of oriental medicine that has been practiced for 1,000's of years. Taichi and Baduanjin have been proven to be effective exercise methods to treat hypertension and improve its complications (10, 11). “Liuzijue” (LZJ) is a traditional exercise that integrates breathing meditation and body movement. It is emphasized to rhythmically use the six syllables of “Xu,” “He,” “Hu,” “Si,” “Chui,” and “Xi” by the nose and mouth. It is an aerobic exercise whose intensity is similar to slow walking (12). It is always exciting to promote blood circulation in traditional Chinese medicine. Modern research showed that this exercise was beneficial to enhance the body's immune function and reduce the level of inflammatory factors (13, 14). Given these functions, we assume that this exercise may be effective in the prevention and treatment of hypertension. In the previous research results, we have achieved some positive conclusions (15), but there was still a lack of sufficient evidence.

The gut microbiota has been recognized to play a key role in the occurrence and development of hypertension (16). The gut microbiota profile significantly differs between hypertensive and healthy subjects (17). In particular, the gut microbiota could release many metabolites such as trimethylamine oxide, bile acids, and short-chain fatty acids into the bloodstream to influence the progression of hypertension (16). Some research have demonstrated that aerobic exercise could improve multiple chronic diseases including hypertension, diabetes, and hyperlipidemia *via* regulating the composition and structure of gut microbiota (18–20). At present, the effect of LZJ exercise on gut microbiota is unclear, and it may be related to the treatment mechanism of hypertension.

In this study, we investigated the effects of 12 weeks of LZJ training on BP levels, vascular endothelial function, and gut microbiota composition in healthy subjects and hypertensive patients. Moreover, we provide new insights into the treatment of hypertension by constructed their potential associations. We hope this study can provide some reference for the application of traditional Chinese exercise in the prevention and treatment of hypertension.

## 2. Methods

### 2.1. Participant filtering

Participants were recruited from Suining Daying County Hospital of Traditional Chinese Medicine, Chengdu Qingyang District traditional Chinese medicine hospital outpatient service, and nearby communities. Patients were first screened over the phone by a research assistant to assess eligibility. Eligible patients were invited for an assessment by a physician, where they were examined and provided detailed written information describing the study. If patients met all inclusion criteria, informed consent was obtained, and they were included in the study.

Male and female patients with hypertension were included according to the Guidelines for Prevention and Treatment of hypertension in China 2010 and European Society for Cardiology/European Society of hypertension guidelines for the management of arterial hypertension criteria (21, 22). Inclusion criteria for hypertensive subjects: (i) Average systolic blood pressure (SBP) ≥ 140 mm Hg and/or diastolic blood pressure (DBP) ≥ 90 mmHg or being treated with antihypertensive drugs (ii) The patient has not taken antibiotics, steroids, and microbial agents in the past month. Further inclusion criteria included basic mobility and the ability to provide informed consent.

Exclusion criteria for hypertensive subjects: (i) Patients with secondary hypertension, such as increased BP or drug-induced hypertension caused by acromegaly, Cushing's syndrome, and other diseases; (ii) In the high-risk or very high-risk state in the stratification of cardiovascular risk level; (iii) Patients with heart, liver, kidney, other organ dysfunction and severe complications such as diabetes; (iv) Patients with a history of intestinal surgery, inflammatory bowel disease, abdominal disease, lactose intolerance, chronic pancreatitis, or other malabsorption diseases.

Inclusion criteria for healthy subjects: (i) Age 45–80, gender unlimited, healthy; (ii) Those who have no habit of overeating and have relatively regular diet; (iii) Have no regular exercise habit at ordinary times (exercise <2 times in a week, and exercise <20 min each time); (iv) In the past month, those who did not take antibiotics, steroids, microbial preparations, blood activating and blood stasis removing herbs, and had no history of diarrhea and other gastrointestinal disorders; (v) Those who have not participated in clinical trials of Taichi, Baduanjin, and other exercise therapies in the past 6 months; (vi) Understand and voluntarily participate in the experimental research, sign the informed consent form, and be able to cooperate to complete the test index detection, with good compliance. Exclusion criteria for healthy subjects: (i) patients with heart, liver, kidney and other organ dysfunction, diabetes, and other serious complications; (ii) People with chronic gastrointestinal diseases; (iii) Long term heavy smokers and drinkers; (iv) Serious trauma and operation history in recent 6 months.

### 2.2. Study planning

This trial is a randomized, controlled, single-blind trial. The sample size is estimated by taking BP as the therapeutic index. It is effective to reduce BP by 5 mmHg in anticipation of exercise intervention (23). The formula for calculating the sample size is based on this (24):


(1) $$n={\left(\frac{(Z_{\text{α}}+Z_{\text{β}})\text{ σ}}{\text{δ}}\right)}^{2}$$

α = 0.05, β = 0.10, 1-β = 0.90, bilateral test, check the normal distribution table *Z*_α_ = 1.96, *Z*_β_ = 1.28, δ = 5, σ = 7.79, substitution formula: *n* = ${\left(\frac{(1.96+1.28)\times 7.79}{5}\right)}^{2}\approx$ 25.

Assuming that the withdrawal or loss rate is 10%, according to the formula: *n*1 = *n*/(1–10%) ≈ 28, 28 subjects are required for each group. There are 56 patients in both groups.

It is estimated that 56 hypertensive subjects will be included in the study. They will be numbered according to the order in which they were included in the study. The random numbers will be obtained through SPSS 24.0 software. The random numbers will be sorted from small to large, and then the subjects will be randomly assigned to different groups (LZJ and AT) according to the random numbers. The researcher in charge of subject recruitment and data statistics did not know the grouping of test subjects, and only made statistical analysis based on the number of subjects.

The study was planned as part of the Key Project of Science and Technology of Sichuan (Grant No. 2020YFS0302). This study followed the principles in the Declaration of Helsinki and has been approved by the Medical Ethics Committee of the Affiliated Hospital of Chengdu University of Traditional Chinese Medicine (approval number: 2020KL-033), and was registered at the China Clinical Trials Registry (http://www.chictr.org.cn/listbycreater.aspx; ChiCTR2200066269).

### 2.3. AT and LZJ training intervention

The intervention of both groups was based on the intensive group behavior intervention. Within 1–2 weeks before the formal training, the LZJ group and AT group were taught by professional doctors to ensure that each subject has mastered the correct movements.

Formal training was conducted under the supervision of researchers. (i) Sports field: a field with a quiet environment and flat ground for training; (ii) Exercise time: 15:00–16:00 on Mondays, Wednesdays, and Fridays; (iii) Exercise frequency: Three centralized pieces of training every week for 12 weeks, a total of 36 times.

The time of concentrated exercise is 1 h, including 5 min of preparatory activities (including chest expansion, stretching, and other exercises to exercise the muscles and joints of the whole body); 50 min of exercise; LZJ and AT have a 5-min relaxation activity after exercise. One set of LZJ exercise is about 12 min and four sets of LZJ movements were performed. A 2-min interval was adopted between two sets and exercises to rest. The AT subjects completed 50 min of AT exercise on the treadmill (IUBU-850T, China), maintaining the heart rate between 40 and 60% of the predicted maximum rate for age. Determined by the following formula: target heart rate = [(220-Age)- rest HR]*(40–60%)+rest HR (25). After 24 min of AT exercise, subjects can rest for 2 min before continuing to exercise.

During the intervention, other factors interfering with the experimental results were excluded. All subjects were not allowed to take any antibiotics, steroids, microbial agents, or traditional Chinese medicine for activating blood circulation and removing blood stasis, as well as other drugs that affect serum inflammatory factors and gut microorganisms. Exercise training such as Taichi and Baduanjin were also prohibited.

### 2.4. Physician-assessed outcomes

All hypertensive subjects were required to follow up once before the intervention (1 week, W1) and after the intervention (12 weeks, W12). Our main purpose was to collect data on the SBP and DBP of hypertensive subjects.

Office SBP and Office DBP were defined by Korotkoff sound phase 1 and phase 5, respectively. The measurement was performed by two experienced physicians and was measured twice. The measurement was considered valid if the difference between the two measurements was <10 mmHg; otherwise, a third measurement was taken, and the average value was used for analysis (26).

The first test of ambulatory blood pressure monitoring (ABPM) was performed before the exercise intervention and used to determine the baseline of hypertensive participants. The second test was performed immediately 48 h after the end of the last exercise intervention to analyze the effect of the exercise intervention. The BP cuff was worn on the non-dominant arm. Subjects were instructed to maintain their customary daily activities, not to exercise, and to relax and unbend the arm during the recording interval for daytime ABPM. An ABPM recording was deemed to be complete if there were at least 14 readings between 6:00 am and midnight and at least six readings between midnight and 6:00 am (27). As recommended by the European Society of Hypertension, daytime SBP and daytime DBP were defined as the mean of all SBP readings and DBP readings, respectively, during the 9:00 am to 9:00 pm window; nighttime SBP and nighttime DBP were defined as the average of all SBP readings and DBP readings, respectively, during the 1:00 AM to 6:00 AM window (28). 24-hour SBP and DBP were defined as the mean of all SBP readings and DBP readings, respectively, over the entire monitoring period.

### 2.5. Serum laboratory measures

Blood samples were drawn at baseline and post-intervention. Blood samples were immediately placed at room temperature for 10 min and then centrifuged at 3,500 rpm for 15 min Preyson Medical Devices) and stored in a freezer at -80°C until they were ready for processing and analysis. Serum nitric oxide (NO), endothelin-1 (ET-1), Interleukins-10 (IL-10), interleukins-6 (IL-6), and matrix metalloproteinase-9 (MMP-9) levels were measured using enzyme-linked immunosorbent assay (ELISA) kits (Geodata Ecosai Biotechnology Co., Ltd) according to the manufacturers instructions and the data were analyzed. All the samples were discarded after the analysis.

### 2.6. Microbial DNA extraction and the 16S rDNA gene sequencing

Fecal samples were collected from participants at baseline and post-intervention. DNA from fecal was isolated by TGuide S96 magnetic bead method soil/feces genomic DNA extraction kit (Tiangen Biochemical Technology Co., Ltd, Beijing, China) and stored at -80°C for future use. The V3-V4 region of the 16S rDNA gene was amplified by the following primers to generate an amplicon library for each sample, 338F (5'-ACTCCTACGGGAGGCAGCA-3') and 806R (5'-GGACTACHVGGGTWTCTAAT-3'). The amplified products were purified with VAHTSTM DNA Clean Beads (Nanjing Nuovizan Biotechnology Co., Ltd, Nanjing, China) and Monarch DNA Gel Extraction (Beijing Hongyue Innovation Technology Co., Ltd, Beijing, China) and quantified by the Qubit 2.0 fluorometer (Invitrogen). The final library was determined whether it meets the requirements of operation by detecting the concentration (Qubit) and size (Agilent 2100); The PacBio Binding Kit was used to combine the library with Primer and Polymerase; The final reaction product was purified by Ampure PB beads and placed on Sequel II sequencer for sequencing.

### 2.7. Bioinformatics analyses

On the PacBio platform, the marker gene was sequenced using SMRT Cell to obtain Circular Consensus Sequencing (CCS) sequences. Then the CCS sequences were filtered, clustered, and denoise to make species annotation.

We analyzed 16S rDNA gene diversity using the Quantitative Insights into Microbial Ecology tool. The Chao, Shannon, Chao1, and ACE diversity indices were used to determine the α-diversity on the operational taxonomic units (OUT) level. Based on the Bray–Curtis distance algorithm, we conducted permutation multiple analysis of variance (PERMANOVA) for gut microbiota data at all levels, and PCoA visualization was conducted at the genus level. The least absolute shrinkage and selection operator (LASSO) was a variable selection method based on the sample data of the penalty method (29). Based on the results of the PERMANOVA analysis, we constructed a LASSO model at the genus level and selected the important species. To screen the baseline characteristic microbiota of hypertensive patients affected by LZJ, we first used LASSO to screen significant taxa in the gut microbiota. Then, based on the logistic regression model, the best variable set was obtained through the internal verification set using 10 levels of cross-validation.

### 2.8. Statistical Analysis

The significance level was determined by appropriate statistical analysis. SPSS 24.0 software (IBM Corp., Armonk, NY) was used for statistical analysis. The normality of all continuous parameters was first checked using the Shapiro-Wilk test. Variables with Gaussian distribution were calculated by *t*-test and expressed as mean ± standard deviation; Otherwise, they will be counted using the Mann-Whitney-*U* and expressed as intermediate values (25–75th percentile).

## 3. Result

### 3.1. Participant characteristics at baseline

**Figure 1:**
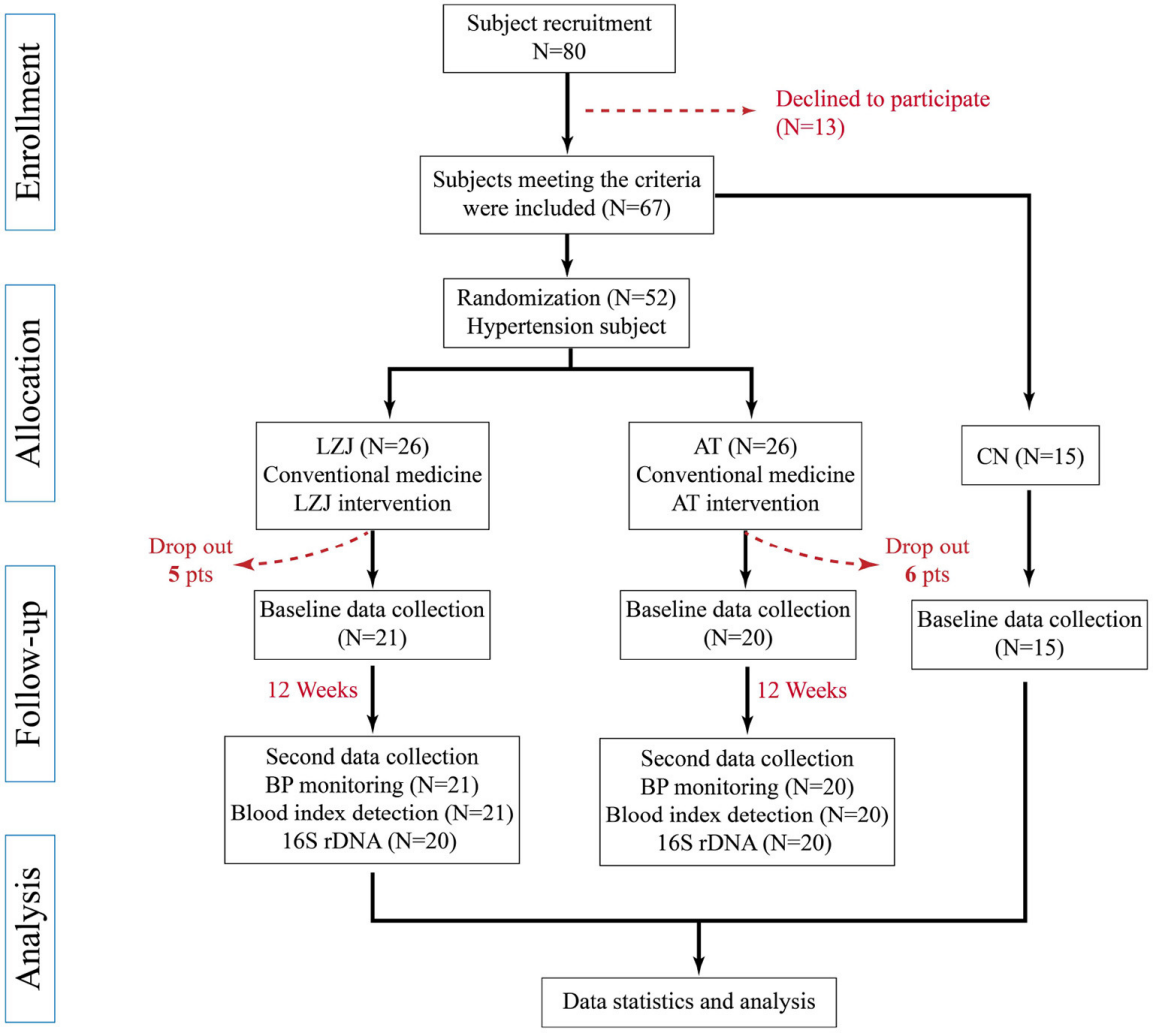
Flow diagram of the study from recruitment to the end of the intervention.

Considering the impact of common antihypertensive drugs on BP, we asked patients not to change the treatment plan and dose of antihypertensive drugs during the test, while controlling important demographic characteristics, such as age and body mass index (BMI). The descriptive statistics of the biodata and anthropometry of the subjects are shown in Table 1. Influenced by various factors, only 52 patients with essential hypertension were finally recruited. During the intervention period, 11 people left the training due to lack of compliance, and only 41 participants with hypertension stayed (Figure 1). A total of 41 pre-hypertensive adults with a mean age for the LZJ and AT groups of 65.19 ± 6.48 and 60.7 ± 8.03 years, respectively, were analyzed. The LZJ group included a total of 21 participants involving 15 (71%) females and 6 (29%) males, while the AT group was made up of a total of 20 participants with 13 (65%) females and 7 (35%) males. The mean BMI of the LZJ group and AT group were 23.88 ± 2.44 and 24.84 ± 2.53 kg/*m*^2^, respectively. Patients of taking calcium channel blocker of the LZJ group and AT group were 20 (95%) and 17 (85%), respectively, and the remaining patients did not take antihypertensive drugs. Furthermore, indexes of BP were not significantly different between LZJ and AT groups at baseline.

**Table 1:** Participant baseline data.

**Index**	**LZJ**	**AT**	**CN**
	**(*N* = 21)**	**(*N* = 20)**	**(*N* = 15)**
Females (%)	71%	65%	87%
Age (years)	65.19 ± 6.48	60.7 ± 8.03	52.93 ± 8.87
BMI (kg/m2)	23.88 ± 2.44	24.84 ± 2.53	21.79 ± 2.09
SBP (mmHg)	153.81 ± 16.57	144.85 ± 18.20	119.13 ± 8.85
DBP (mmHg)	85.19 ± 12.15	88.9 ± 10.33	77.27 ± 10.75
HR (BPM)	76.00 ± 12.43	70.05 ± 10.49	67.44 ± 11.40
24 h ABPM			
SBP (mmHg)	142.57 ± 16.18	130.6 ± 14.75	112.73 ± 8.45
DBP (mmHg)	82.52 ± 9.11	84.55 ± 11.90	67.47 ± 7.09
Day-time ABPM			
SBP (mmHg)	143.71 ± 17.13	132.15 ± 16.04	113.2 ± 7.97
DBP (mmHg)	85.76 ± 10.87	87.05 ± 13.44	67.8 ± 7
Night-time ABPM			
SBP (mmHg)	140.67 ± 18.47	127 ± 16.14	109.07 ± 13.58
DBP (mmHg)	77.95 ± 8.88	79.55 ± 11.51	65.8 ± 9.14
ET-1 (pg/mL)	9.31 ± 10.39	9.88 ± 12.61	8.48 ± 12.75
NO (pg/mL)	3.81 ± 5.82	2.32 ± 0.87	2.99 ± 0.45
MMP-9 (pg/mL)	3,639.94 ± 2,690.04	4,382.04 ± 2,767.32	1,429.51 ± 1,707.32
IL-6 (pg/mL)	4.00 ± 2.10	2.99 ± 1.17	2.554 ± 0.81
IL-10 (pg/mL)	4.81 ± 2.85	7.016 ± 9.12	6.438 ± 4.75
Calcium channel blocker(%)	95%	85%	0
Not taking antihypertensive drug(%)	5%	15%	100%

### 3.2. The effects of LZJ exercise for BP in hypertensive patients

**Figure 2:**
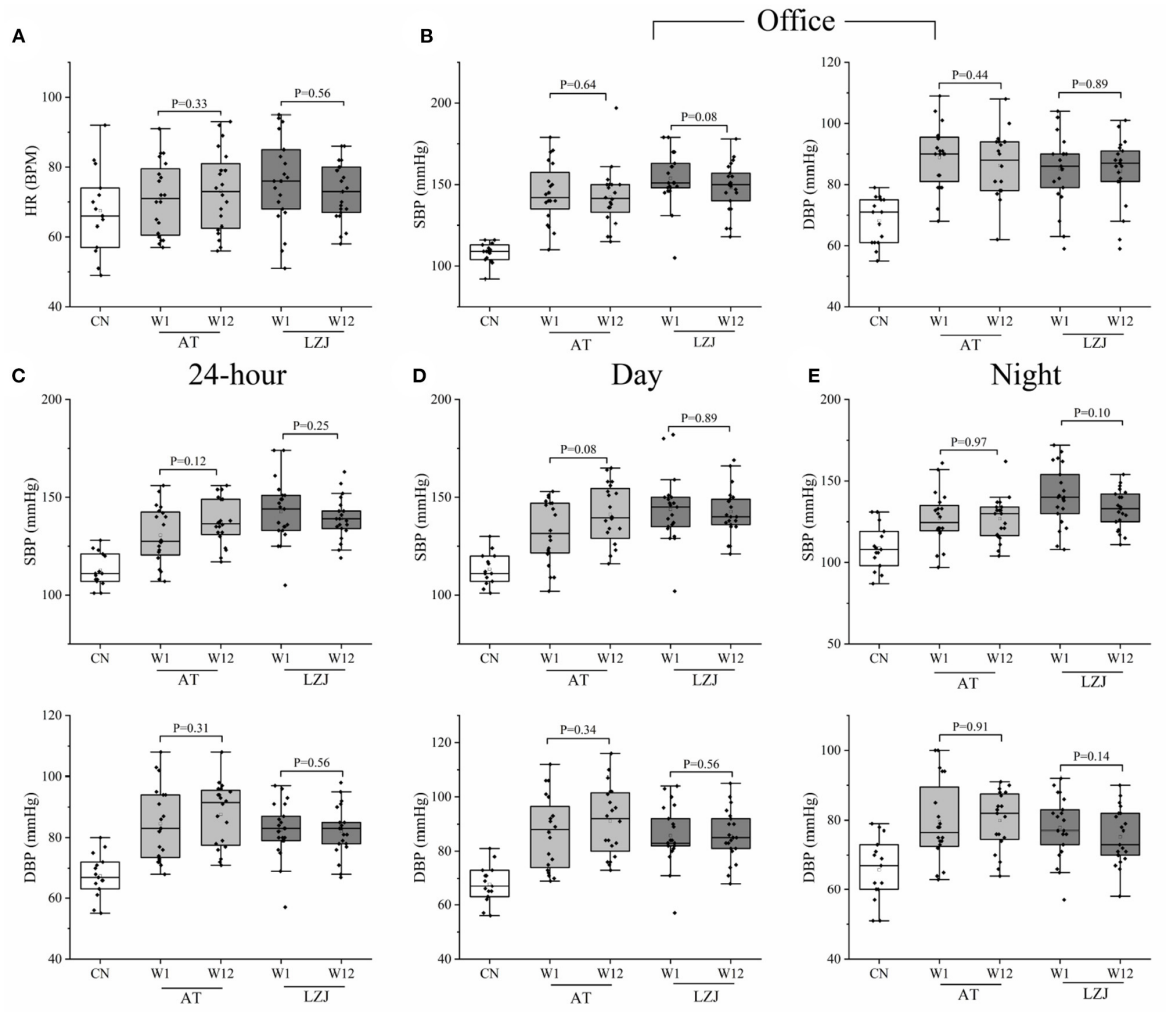
Changes in clinical indicators between W1 and W12. **(A)** HR changes after the intervention; **(B)** Changes in-office blood pressure after the intervention; **(C–E)** Changes in ambulatory blood pressure after the intervention. The line within the boxplot indicates the median. Whiskers are drawn to a minimum and maximum values, but not further than 1.5 × IQR. Differences in exercise intervention for 12-week changes from baseline of laboratory indexes were tested by analysis of *t*-test or Mann-Whitney-*U* test based on whether the data were normally distributed.

After 12 weeks of training, the HR level of the LZJ group showed a downward trend compared with that of AT group (Figure 2A, Supplementary Tables 1, 2). For office BP, both AT and LZJ could slightly reduce SBP and DBP levels after 12 weeks of intervention (Figure 2B). In addition, LZJ excise has a certain positive impact on 24-h SBP and night SBP, with the *P*-values of 0.25 and 0.10, respectively. For the Day SBP, there was no obvious change after the intervention (Figures 2C–E). For other indicators, AT and LZJ have no beneficial regulatory effect. According to our preliminary statistics, there was no significant change in BP before and after AT or LZJ intervention (Supplementary Tables 1, 2).

**Figure 3:**
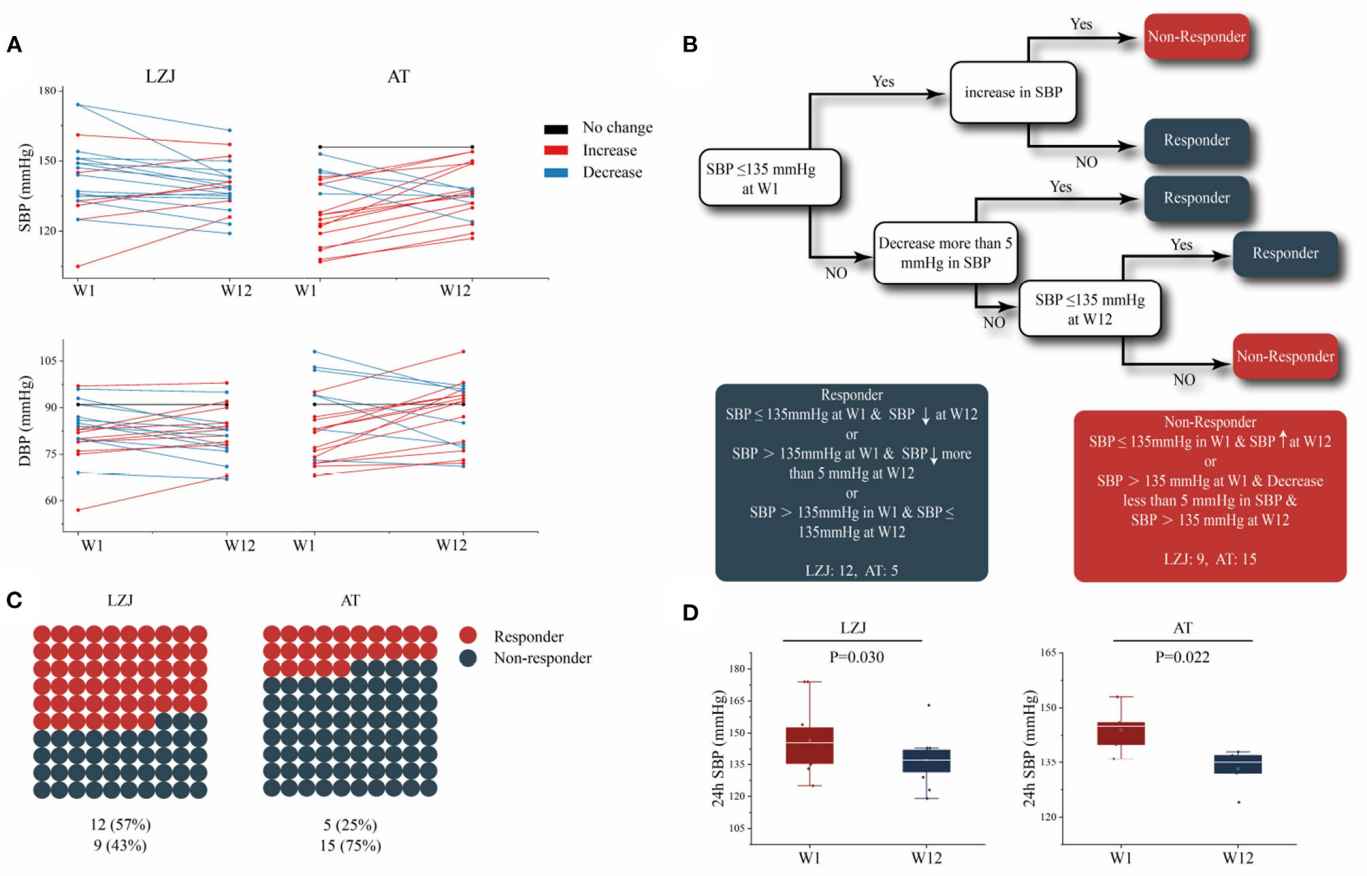
The effect of AT and LZJ exercises on hypertensive. **(A)** Lines show individual participant trajectories at the 24 h SBP. **(B)** Decision tree for the determination of BP responders and BP non-responders. According to the changes of 24 h-SBP in individuals before and after intervention (W1–W12), hypertensive patients were divided into BP responders and BP non-responders. BP responders: (i) SBP was lower or equal to 135 mmHg at W1, and decreased at W12; (ii) SBP is higher than 135 mmHg at W1 and decreases more than 5 mmHg at W12; (iii) SBP is higher than 135 mmHg at W1 but lower than or equal to 135 mmHg at W12. BP non-responders: (i) SBP is lower than or equal to 135 mmHg at W1, but SBP rises at W12; (ii) SBP is higher than 135 mmHg at W1, SBP decrease is not more than 5 mmHg and SBP is still higher than 135 mmHg at W12; **(C)** The proportion of BP responders in LZJ and AT groups, with red circles representing the BP responder and blue representing the non-BP responder; **(D)** The change of 24 h SBP in BP responders; The line within the boxplot indicates the median. Whiskers are drawn to minimum and maximum values, but not further than 1.5 × IQ.

Since individual differences in hypertensive patients may affect the intervention effect of LZJ or AT, we analyzed the clinical data of each subject separately. The results showed that only 5 (25%) subjects' BP decreased after AT intervention, 1 (5%) subject's BP was consistent with that before the intervention, and the other 14 (70%) subjects' BP increased. After LZJ intervention, the BP of 15 (71%) subjects decreased, and those of 6 (29%) subjects increased (Figure 3A). The proportion of hypertensive patients with decreased BP in the LZJ group was obviously higher than that in AT group. These results indicated that the effect of exercise intervention on lowering BP was affected by individual differences, and it was only applicable to some specified= hypertensive patients. In addition, LZJ was more suitable for ordinary hypertensive patients than AT.

Based on the previous study (23), we artificially constructed a decision tree to stratify hypertensive patients (responder or non-responder) according to their ABPM (30) (Figure 3B). Twelve subjects in LZJ group were classified as BP responders, which proved that LZJ could improve the BP of 57% of hypertensive subjects. There were only 5 (25%) BP responders in AT group, which had a much lower impact on hypertensive subjects than LZJ exercise (Figure 3C). In addition, the changes in SBP of BP responders before and after intervention were statistically analyzed. It was found that the SBP of BP responders after exercise intervention (AT and LZJ) decreased significantly, with *P* = 0.022 and *P* = 0.030, respectively (Figure 3D).

According to the results, AT and LZJ can significantly reduce BP, but only for some proportion of hypertensive patients. Compared with AT, LZJ exercise affected higher proportion of hypertensive patients by reducing the BP levels, meaning that LZJ is more suitable for clinical promotion as an effective intervention strategy for hypertension.

### 3.3. The changes of NO, ET-1, MMP-9, IL-6, and IL-10 levels in BP responders

**Figure 4:**
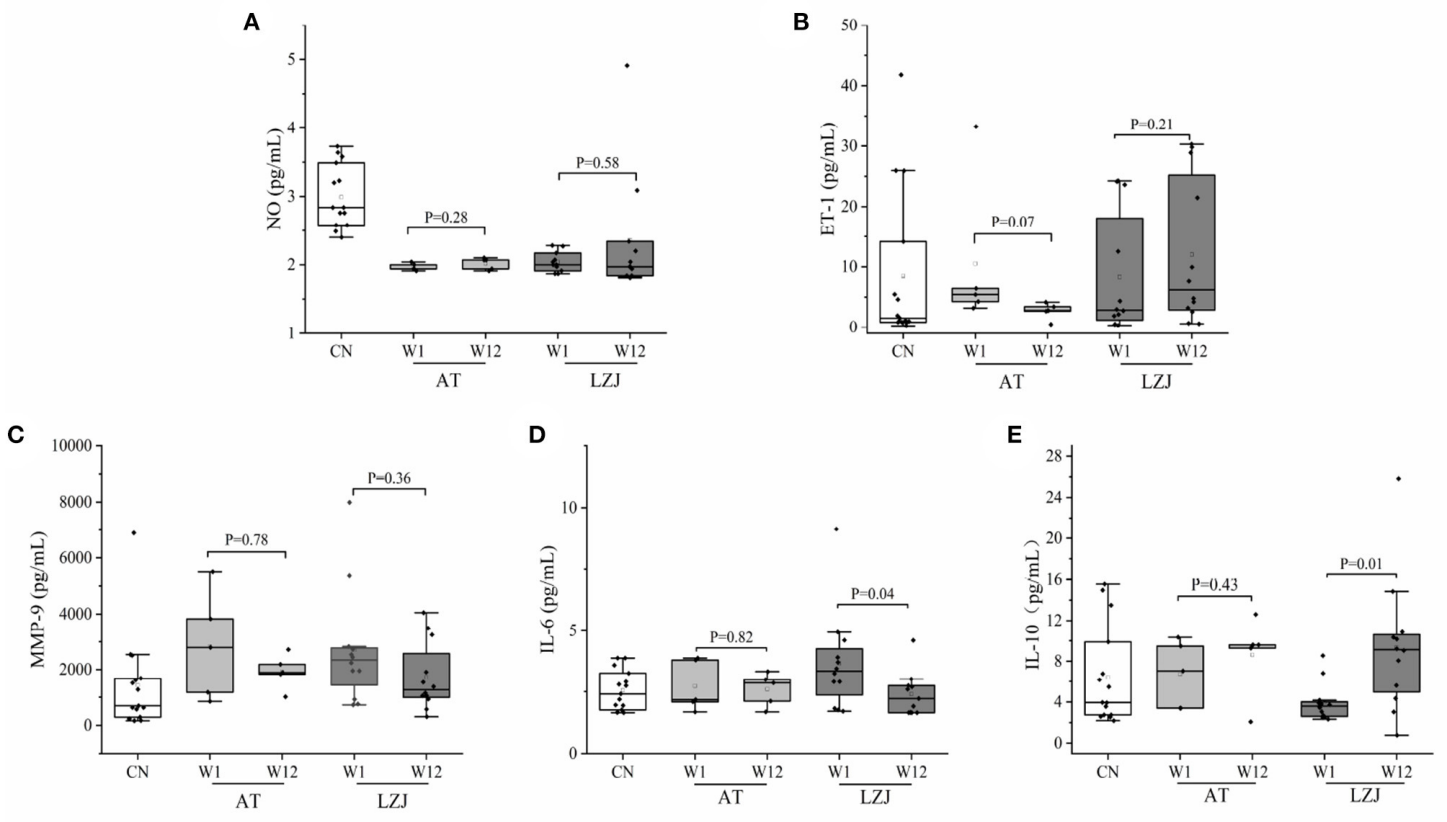
BP responder specific changes in the vascular endothelial function and immune response. **(A–E)** LZJ and AT caused changes in ET-1, NO, MMP-9, IL-6, and IL-10 in hypertensive patients persisting 3 months later. The line within the boxplot indicates the median. Whiskers are drawn to minimum and maximum values, but not further than 1.5 × IQ. Differences in exercise intervention for 12-week changes from baseline of laboratory indexes were tested by analysis of *t*-test or Mann-Whitney-*U* test based on whether the data were normally distributed.

NO and ET-1 are the important cytokines to characterize endothelial function (31, 32). Our results showed that AT intervention could slightly reduce the ET-1 level of BP responders (*P* = 0.07), while had no effect on NO level (*P* = 0.28). LZJ intervention had no effect the ET-1 and NO levels of BP responders, with the *P*-values of 0.21 and 0.58, respectively (Figures 4A, B, Supplementary Tables 3, 4).

High expression of MMP-9 was a typical indication of hypertensive patients, which further caused the activation of endothelial cells, vascular endothelial dysfunction, and immune homeostasis imbalance (33–35). Both AT and LZJ interventions reduced the concentration of MMP-9 in BP responders, but the difference was not significant (Figure 4C). Additionally, after exercise intervention (LZJ and AT), the concentration of inflammatory factor IL-6 decreased, and the concentration of anti-inflammatory factor IL-10 increased (Figures 4D, E). In particular, LZJ significantly inhibited the expression of IL-6 (*P* = 0.04) and promoted the differentiation of IL-10 (*P* = 0.01), indicating that LZJ exercise could significantly improve the immune homeostasis of BP responders.

In conclusion, exercise intervention had little effect on the vascular endothelial function of BP responders in this experiment. Unexpectedly, exercise intervention had a great effect on the immune balance of BP responders. In particular, LZJ could significantly improve the immune homeostasis of hypertensive patients by regulating the concentrations of IL-6 and IL-10. Previous studies have shown that exercise intervention could reduce hypertension complications by regulating immune homeostasis and lowering BP (36, 37). The function of LZJ in reducing responders' BP may be related to the regulation of immune homeostasis.

### 3.4. The effects of LZJ exercise on gut microbiota in hypertensive patients

Numerous studies have shown that gut microbiota disorders were associated with hypertension (11). After clustering at 97% similarity level, 391 effective OTUs sequences were obtained, and they were used to further analyze the structure and composition of gut microbiota. A total of nine phyla, 15 classes, 25 orders, 50 families, 154 genera, and 210 species of gut microbes were successfully annotated for subsequent analyses.

**Figure 5:**
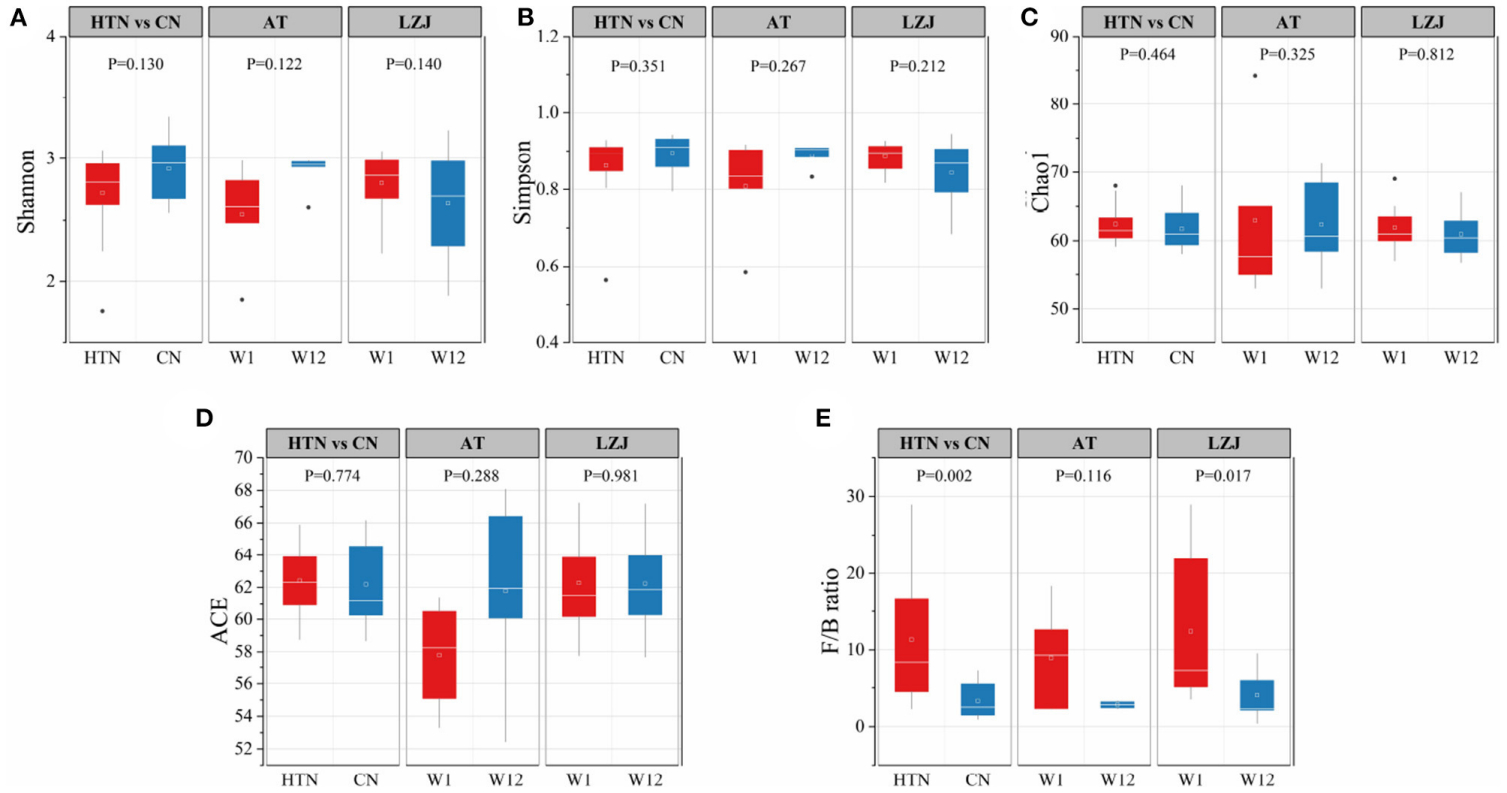
Differences in the abundance and structure of gut microbiota. **(A)** Shannon index; **(B)** Simpson index; **(C)** Chao1 index; **(D)** ACE index; **(E)** F/B ratio; The line within the boxplot indicates the median. Whiskers are drawn to minimum and maximum values, but not further than 1.5 × IQ.

Firstly, we used α-diversity indexes (Shannon, Simpson, Chao1, and ACE) to evaluate the changes in the microbial community structure of BP responders after exercise intervention. Figure 5 has shown no significant difference in the diversity and abundance of gut microbiota between hypertensive patients and healthy people. Similarly, there was no significant difference in diversity and abundance of gut microbiota before and after LZJ and AT intervention.

However, there was a significant difference in Firmicutes/Bacteroidetes (F/B) ratio between hypertension and healthy people. As seen in Figure 5E, the F/B ratio of healthy people was significantly lower than that of hypertension patients(*P* = 0.002). The F/B ratio has been used extensively as a proxy for broad shifts in microbial community structure from mammalian feces (38). After exercise intervention, the F/B ratio of BP responders also decreased. In particular, the F/B ratio of BP responders was significantly decreased after LZJ intervention (*P* = 0.017), indicating that this exercise could improve the dysbiosis of gut microbiota caused by the elevation of BP level.

**Figure 6:**
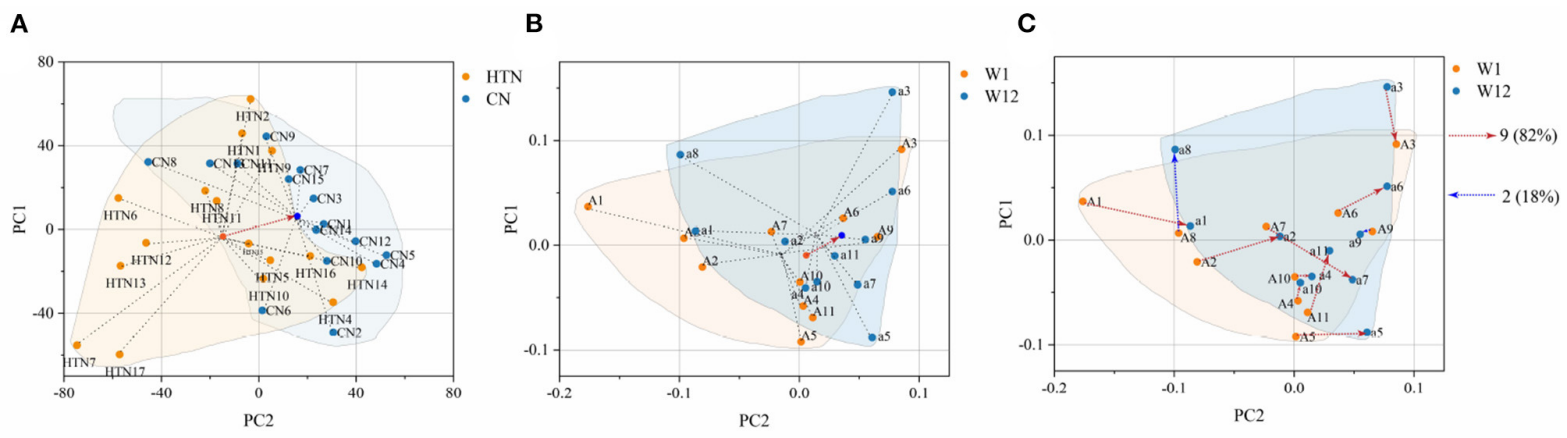
Change trend of gut microbiota structure. **(A)** PCoA analysis between hypertensive and healthy populations; **(B)** PCoA analysis before and after LZJ intervention; **(C)** Change trajectory of BP responders affected by LZJ. The black dotted line is the connection between samples in the group. The red arrow is the displacement trajectory in the same direction as that of the healthy population, and the blue arrow is the displacement trajectory in the opposite direction.

Compared to α-diversity, the β-diversity index was most sensitive to the structural difference of gut microbiota. Regarding β-diversity, the Bray-Curtis metric was in general the most sensitive to observing differences between groups (39). We conducted PERMANOV analysis based on the Bray-Curtis distance and found that there were significant differences in gut microbiota structure between hypertensive patients and healthy people at all levels, especially at the genus level (*P* = 0.0001, Supplementary Table 5). Therefore, we analyzed the structural differences of gut microbiota among different groups based on PCoA at the genus level. The result showed that the healthy population and hypertensive patients presented two independent clusters with difference gut microbiota structures (Figure 6A). After LZJ intervention, the midpoint of the connecting line between hypertensive patients shifted toward the healthy population (Figure 6B), indicating that LZJ could convert the gut microbiota structure of hypertensive patients into the healthy population. We further connected the coordinates of hypertensive patients at W1 and W12. The results showed that the gut microbiota of 82% of BP responders tended to move in the healthy population direction after LZJ intervention (Figure 6C). These results also proved that LZJ had a beneficial effect on the gut microbiota structure for most hypertensive patients.

**Figure 7:**
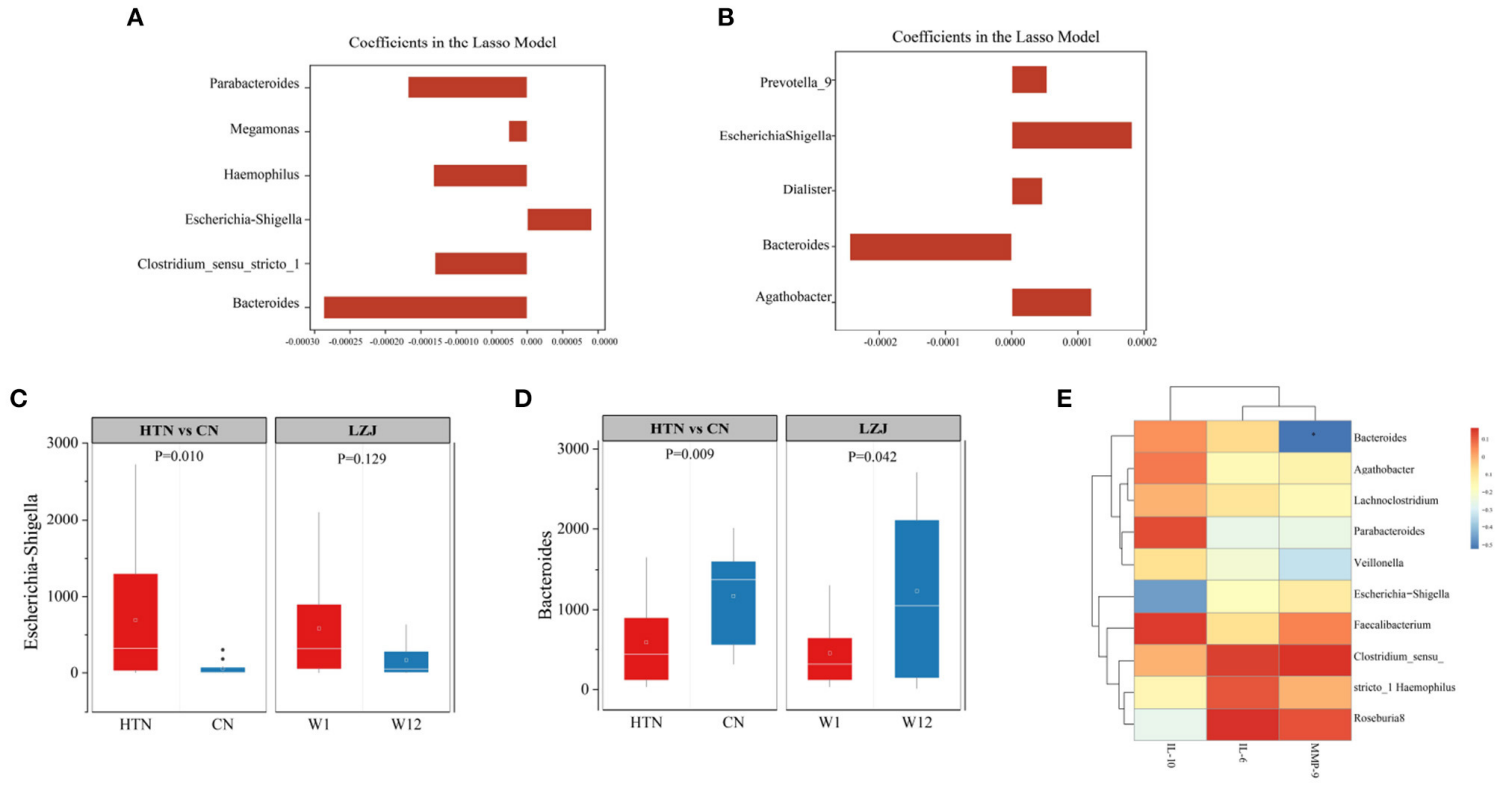
Screening of differential microbiota after LZJ intervention. **(A)** Screening of differential microbiota of BP responders after LZJ intervention; **(B)** Screening of differential microbiota between BP responders and healthy people; **(C, D)** Abundance of Escherichia-Shigella, Bacteroidetes. The bar plots represent the regression in a model with binary output for every feature. Whiskers are drawn to minimum and maximum values, but not further than 1.5 × IQR; **(E)** Correlation analysis on immune stability and gut microbiota structure.

Subsequently, we used the LASSO model to screen the most significantly changed microbiota of hypertensive subjects after LZJ intervention at the genus level. The result showed that Bacteroides, Clostridium_sensu_stricto_1, Escherichia-Shigella, Haemophilus, Megamonas, and Parabacteroides were the main characteristic bacteria genus (Figure 7A). Among them, Bacteroidetes and Escherichia-Shigella were also the difference microbiota between hypertensive subjects and healthy people (Figure 7B). These bacteria have been proven to play a key role in the process of hypertension (40, 41). The previous study (23) have proven that Bacteroidetes can produce the short-chain fatty acids and activate their related receptors, thus lowering the BP level of rats. In addition, the increase in the abundance of this Escherichia-Shigella is believed to be related to hypertension (42). It can activate the NLRP inflammasomes, release pro-inflammatory factors and induce inflammation and is highly concentrated in hypertensive patients (43).

According to our results, LZJ exercise reversed the abundance of both taxa in hypertension patient, especially Bacteroidetes (*P* = 0.042, Figures 7C, D). We speculate that these two colonies may be the key colonies for LZJ exercise to improve hypertension symptoms.

Since LZJ exercise significantly reduced BP, improved the immune stability and gut microbiota structure of BP responders. We conducted correlation analysis on immune stability and gut microbiota structure to obtain more reliable results. The results showed that there was a significant negative correlation between Bacteroides and MMP-9, and there was positive correlation between Bacteroides and immunosuppressive factor of IL-10 With respect to Escherichia-Shigella, this bacteria has an apparent negative correlation with IL-10 (Figure 7E). These results proved that there is an interaction between immune factors and gut microbiota, which may be the potential mechanism of LZJ exercise to lower BP level.

### 3.5. Baseline indicators predicting LZJ BP responders

As mentioned above, 57% of hypertensive patients have significantly decreased their BP level after LZJ intervention. We sought to understand whether the factors underlying LZJ's successful intervention in hypertensive patients could be predicted at baseline. Considering that there was no significant difference in the baseline levels of ET-1, NO, MMP-9, IL-6, and IL-10 between BP responders and non-responders before the intervention (Supplementary Table 6). The possibility of laboratory indicators (ET-1, NO, MMP-9, IL-6, and IL-10) as baseline characteristics to explain the improved prognostic outcomes of BP was excluded.

**Figure 8:**
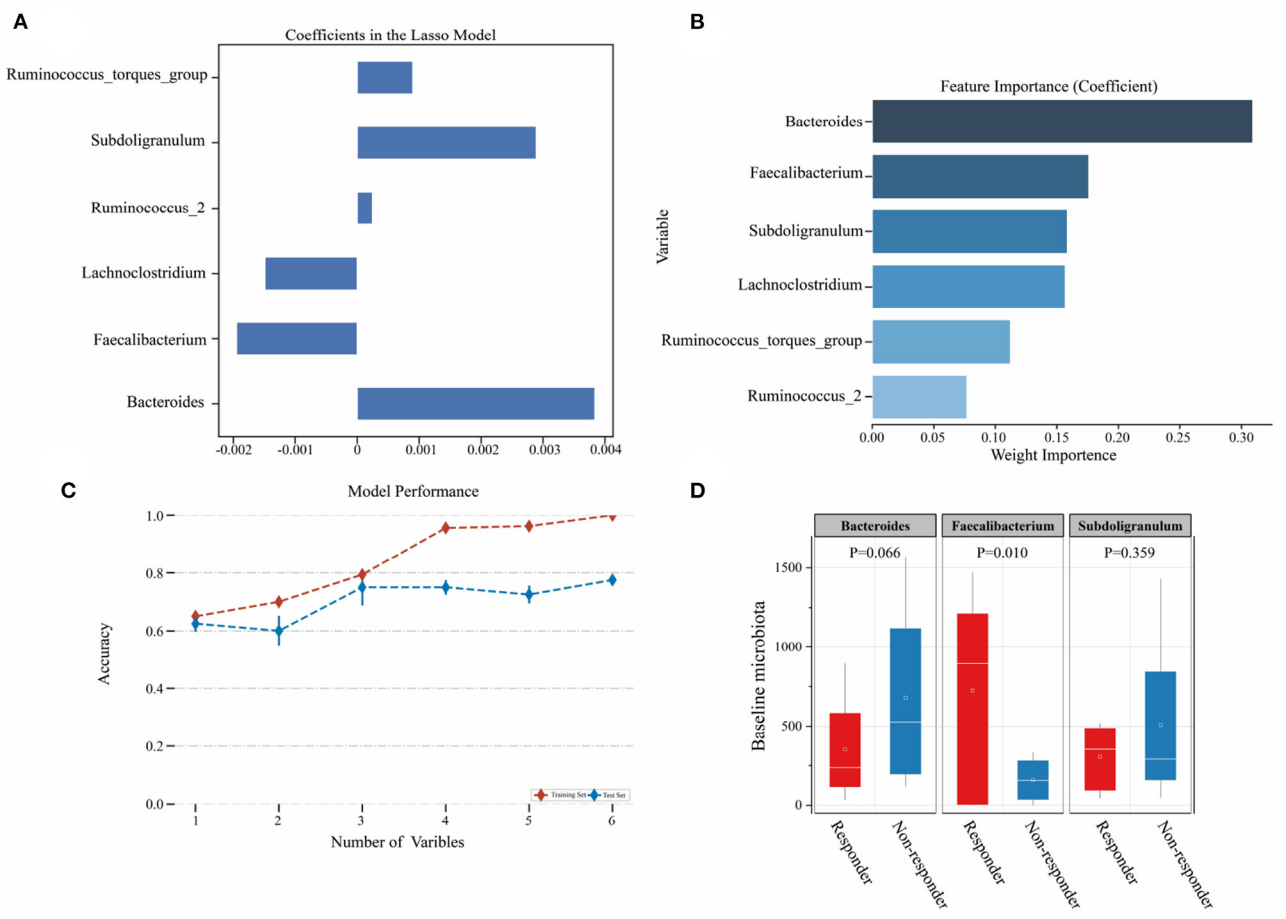
Screening of baseline characteristics of the gut microbiota in BP responders. **(A)** Screening of characteristic microbiota of BP responders at baseline; **(B)** Ranking of feature importance; **(C)** Model evaluation; **(D)** Baseline characteristic flora abundance; Whiskers are drawn to a minimum and maximum values, but not further than 1.5 × IQR.

Subsequently, we tried to explore the feasibility of microorganisms as predictors of treatment effects in hypertensive patients. Based on 16S rDNA baseline data, we screened six significant response-specific taxa through the LASSO model (Figure 8A). Then we used logistic regression to score the variable set and Bacteroides, Faecalibacterium, and Subdoligranulum were selected as the most important variable set through cross-validation (Accuracy = 0.794; Figures 8B, C). These variables were involved in the construction of predictors of treatment effects in the hypertensive patients' model, and we aim to screen the applicable hypertensive audience for LZJ through this model. To understand the baseline characteristics of BP responders in more detail, we visualized the three taxa and found that low levels of Bacteroides and Subdoligranulum, and high levels of Faecalibacterium were the characteristics of BP responders at the baseline stage (Figure 8D), which provided a certain reference basis for clinical screening of LZJ applicable populations. In addition, Bacteroidetes was not only the baseline microbiota of BP responders but also the key microbiota for LZJ to intervene in hypertension.

## 4. Discussion

LZJ was a traditional exercise method that originated in ancient China. Its main feature was to integrate breathing meditation and body movement, to promote the circulation of qi and blood. Previous studies have shown that LZJ can enhance immune function and has a certain antihypertensive effect, but there is still insufficient evidence (15). Therefore, we designed this study to investigate the regulatory effect of LZJ on patients with primary hypertension and to detect the changes in BP related cytokines and gut microbes before and after the intervention, in order to find the potential functional mechanism.

In this study, we found that exercise training (LZJ and AT) could selectively reduce the BP level of hypertensive patients, and showed a certain positive effect on hypertensive patients. Through decision tree screening, LZJ intervention has a beneficial impact on 57% of hypertensive patients, while only 25% of hypertensive patients are beneficially affected by AT. Therefore, compared with AT exercise, LZJ intervention was more suitable for hypertensive patients and has a more positive clinical effect.

Immune homeostasis plays a key role in the pathogenesis of hypertension. The immune imbalance will release a large number of inflammatory mediators (MMP-9, IL-6, etc.), stimulate vascular fibrosis, and end organ damage (40). Both patients with essential hypertension and hypertensive animal models showed elevated levels of IL-6 in systemic circulation (41, 44). In our study, LZJ intervention significantly inhibited the high expression of IL-6 and promoted the differentiation of the anti-inflammatory factor IL-10, indicating that LZJ may achieve the goal of lowering BP by promoting immune homeostasis.

In addition, the balance of gut microbiota is an important factor in BP homeostasis, although its mechanism is complex and partly unknown (17). There is convincing evidence that Firmicutes and Bacteroidetes account for most types of total bacteria in the healthy intestine, and their proportion is generally considered a relative indicator of gut microbiota health or ecological imbalance (45). The F/B ratio of hypertension patients increased, and our experimental results were consistent with this conclusion (42). LZJ significantly reduced the F/B ratio and converted the gut microbiota structure of BP responders to that of healthy people. Compared with patients with hypertension, LZJ reduced the relative abundance of Escherichia-Shigella, which is believed to be related to hypertension (45). After LZJ intervention, the abundance of Escherichia-Shigella decreased, and the stability and inflammation of the host intestinal structure improved. In addition, Bacteroides was also the difference in microbiota between hypertensive patients and healthy people. The level of Bacteroides increased significantly after LZJ intervention. As a beneficial microbial community, Bacteroides play an important role in the systemic immune response (46, 47). These microbial communities may be closely related to the hypotensive effect of LZJ.

To explore the causes of baseline LZJ lowering BP, we used LASSO and logistic regression models to screen the baseline characteristic microbiota of BP responders at the genus level. The results showed that the baseline characteristic colonies of BP responders were: Bacteroides, Faecalibacterium, and Subdoligranulum. These characteristic microbiotas are all related to the metabolism of short-chain fatty acids (48–51). As a metabolite of gut microbiota, short-chain fatty acids is usually used to indicate the reduction of cardiovascular adverse events and risks (52) and is widely considered a potential mediator involved in regulating BP and inhibiting chronic inflammation (26). In particular, lower Bacteroides were the baseline feature of BP responders, and the abundance increased significantly after LZJ intervention. Therefore, Bacteroides should be paid enough attention to in patients with hypertension.

To sum up, our experiment proves that LZJ may be more effective than conventional AT in treating hypertension. This exercise could be used as an auxiliary treatment scheme for hypertension and is suitable for extensive clinical promotion. Specifically, compared with AT, LZJ was more generally applicable to hypertensive patients and showed a higher proportion of BP responders. It has a more significant effect on the improvement of inflammation and gut microbiota, which could reduce the abundance of harmful bacteria and increase the abundance of probiotics in the gut microbiota, especially Escherichia-Shigella and Bacteroides. But, the relatively low number of patients may be regarded as a limitation, and experiments in more and larger studies are also needed in the later stage to comprehensively determine the antihypertensive effect of LZJ.

## Data availability statement

The original contributions presented in the study are included in the article/Supplementary material, further inquiries can be directed to the corresponding author.

## Ethics statement

The studies involving human participants were reviewed and approved by the Medical Ethics Committee of the Affiliated Hospital of Chengdu University of Traditional Chinese Medicine (approval number: 2020KL-033). The patients/participants provided their written informed consent to participate in this study.

## Author contributions

LX and LQ designed and supervised the entire study. SW, CZ, NL, TD, LX, JW, and LQ performed the experiments. SW and CZ prepared the manuscript. LQ and SW were involved in the data analysis and manuscript revision. All authors contributed to the article and approved the submitted version.
