# Supplementary material for: Liuzijue training improves hypertension and modulates gut microbiota profile
Source: Front Cardiovasc Med. 2023 Jan 25;10:1075084. doi: 10.3389/fcvm.2023.1075084 (PMC9905721; doi:10.3389/fcvm.2023.1075084)
Supplement: Supplementary file 1 [file Data_Sheet_1.PDF]

Table.S1 LZJ clinical index analysis results

Index	W1 (n=21)	W12 (n=21)	P	Method
Office SBP, mean(±SD)	156.00±11.99	148.24±14.99	0.08	t-test
Office DBP mean(±SD)	86.52±10.31	86.91±7.37	0.89	t-test
HR, mean(±SD)	76.00±12.13	72.76±8.18	0.33	t-test
24hSBP, median[IQR]	144.00[135.00,151.00]	139.00[135.00,141.00]	0.25	Mann-Whitney-U
24hDBP, mean(±SD)	83.76±6.83	82.19±6.40	0.56	t-test
Day SBP, mean(±SD)	141.69±7.85	141.26±10.50	0.89	t-test
Day DBP, mean(±SD)	87.05±8.47	85.67±9.31	0.63	t-test
Night SBP, mean(±SD)	140.67±18.03	132.62±11.73	0.10	t-test
Night DBP, mean(±SD)	78.91±7.30	75.24±7.95	0.14	t-test

Table.S2 AT clinical index analysis results

Index	W1 (n=20)	W12 (n=20)	P	Method
office SBP, mean(±SD)	144.85±17.74	142.15±17.59	0.64	t-test
office DBP, mean(±SD)	88.90±10.06	86.30±10.50	0.44	t-test
HR, mean(±SD)	71.05±10.14	73.10±11.35	0.56	t-test
24hSBP, mean(±SD)	130.60±14.38	137.25±11.39	0.12	t-test
24hDBP, median[IQR]	83.00[74.00,94.00]	92.00[78.00,95.00]	0.31	Mann-Whitney-U
Day SBP, mean(±SD)	132.15±15.63	141.20±15.06	0.08	t-test
Day DBP, mean(±SD)	87.05±13.10	91.10±12.49	0.34	t-test
Night SBP, mean(±SD)	127.00±15.74	126.80±13.07	0.97	t-test
Night DBP, mean(±SD)	79.55±11.22	79.90±8.12	0.91	t-test

Table.S3 AT laboratory index analysis results

	W1(n=5)	W12(n=5)	P	Method
ET-1, mean (SD)	4.55(1.23)	2.71(1.23)	0.07	t-test
N0, mean (SD)	1.97(0.05)	2.02(0.08)	0.28	t-test
MMP-9, mean (SD)	2110.98(1087.5)	1932.77(553.62)	0.78	t-test
IL-6, mean (SD)	2.73(0.93)	2.60(0.61)	0.82	t-test
IL-10, mean (SD)	6.74(2.93)	8.63(3.50)	0.43	t-test

Table.S4 LZJ laboratory index analysis results

Index	W1 (n=12)	W12 (n=12)	P	Method
ET-1, median [IQR]	2.97[1.88,12.53]	7.64[3.20,21.46]	0.21	Mann-Whitney-U
NO, mean (SD)	2.4(0.13)	2.00(0.15)	0.58	T-test
MMP-9, mean (SD)	2199.46(1188.86)	1726.66(1162.63)	0.36	T-test
IL-10, median [IQR]	3.74[2.69,3.86]	9.05[4.35,10.17]	0.01	Mann-Whitney-U
IL-6, median [IQR]	3.25[2.73,3.71]	2.60[1.66,2.73]	0.04	Mann-Whitney-U

Table.S5 PERMANOVA Analysis

Taxa	Pairs	Df	Sums Of Sqs	F.Model	R2	P.value	P.adjusted	sig
Phylum	HTN vs CN	1	0.211	4.549	0.14	0.016	0.016	*
Order	HTN vs CN	1	0.271	3.217	0.103	0.013	0.013	*
Class	HTN vs CN	1	0.308	3.922	0.123	0.01	0.01	*
Family	HTN vs CN	1	0.523	2.93	0.095	0.002	0.002	**
Genus	HTN vs CN	1	0.688	2.864	0.093	0.001	0.001	***
Species	HTN vs CN	1	0.698	2.627	0.086	0.002	0.002	**
OTU	HTN vs CN	1	0.708	2.315	0.076	0.002	0.002	**

Sign in. codes: P≤0.001 ‘***’ P≤0.01 ‘**’; P≤0.05 ‘*’

Table.S6 LZJ baseline difference analysis

Index	Responder(n=12)	Non-Responder (n=9)	P	Method
SBP, median[IQR]	151[148,170]	151 [148,161]	0.70	Mann-Whitney-U
DBP, mean(±SD)	86.250(±12.657)	86.889(±5.858)	0.90	t-test
HR, mean(±SD)	78.67(±11.85)	72.44(±11.58)	0.27	t-test
24h-SBP, mean (±SD)	146.58(±14.66)	137.22(±15.65)	0.20	t-test
24-DBP, median [IQR]	84[82,86]	79 [76,91]	0.32	Mann-Whitney-U
Day-SBP, mean (±SD)	141.5(±6.78)	142.78(±9.02)	0.73	t-test
Day-DBP, mean (±SD)	86.25(±8.20)	85.11(±13.13)	0.82	t-test
Night-SBP, mean(±SD)	144.83(±16.44)	131.50(±13.47)	0.08	t-test
Night-DBP, mean(±SD)	80.21(±4.47)	76.44(±11)	0.38	Welch's t-test
ET-1, median[IQR]	2.97[1.88,12.53]	2.87[1.53,9.55]	0.86	Mann-Whitney-U
NO, median[IQR]	2[1.97,2.07]	2[2,2]	0.70	Mann-Whitney-U
MMP-9, median[IQR]	2447.12 [1956.23,2447.12]	6616.12 [1640.78,7577.28]	0.06	Mann-Whitney-U
IL-6, mean (±SD)	3.2(±1.01)	3.45(±0.36)	0.46	Welch's t-test
IL-10, mean (±SD)	3.25(±0.59)	4.06(±1.11)	0.06	t-test

Table.S7 The drug information of participants

Drugs	LZJ	AT
Nifedipine GITS 30ml/d	2	2
Nifedipine GITS 30ml/d+Amlodipine Besylate Tablets 5mg/d	0	1
Nifedipine Tablets 10mg/d	3	1
Nifedipine Tablets 5mg/d	0	2
Lacidipine Tablets 4mg/d	1	2
Lacidipine Tablets 2mg/d	1	1
Nitrendipine Tablets 10mg/d	3	2
Amlodipine Besylate Tablets 5mg/d	9	5
Felodipine Tablet 2.5mg/d	1	1
Not taking antihypertensive drugs	1	3
